# An Unusual Clinical Presentation of Immunoglobulin A Vasculitis in an Elderly Patient

**DOI:** 10.7759/cureus.10946

**Published:** 2020-10-14

**Authors:** Akudo U Anokwute, Olusegun Bakare, Chukwunonye O Ogbuji, Nkechi C Arinze

**Affiliations:** 1 Internal Medicine, Brookdale University Hospital Medical Center, Brooklyn, USA; 2 Internal Medicine, Thomas Jefferson University, Philadelphia, USA; 3 Internal Medicine, Baylor University, Waco, USA; 4 Internal Medicine/Community Medicine, Mercer University School of Medicine, Macon, USA

**Keywords:** rare association, leukocytoclastic vasculitis, skin biopsy, deep vein thrombosis (dvt), ecchymoses, arthritis and orthopaedic rheumatology, cellulitis, henoch schönlein purpura, immunoglobulin a vasculitis

## Abstract

Immunoglobulin A (IgA) vasculitis (IgAV), also known as Henoch-Schonlein purpura, is an IgA-mediated leukocytoclastic vasculitis predominantly affecting the gastrointestinal tract, kidneys, and skin. IgAV appears to be more common in children and is relatively rare in the adult population. We describe a case of a 72-year-old man who presented with bilateral lower extremity swelling, nausea, vomiting, abdominal pain, and weight loss, in which a diagnosis of IgAVs was established with a skin biopsy. This case highlights a rare and unusual clinical presentation of IgAV in an older patient and the need for prompt diagnosis and treatment.

## Introduction

Immunoglobulin A (IgA) vasculitis (IgAV), formerly called Henoch-Schönlein purpura [1], is the most common form of systemic vasculitis in children. IgAV is usually self-limited, and in most cases, present in pediatric patients. The major clinical findings include palpable purpura in patients with neither thrombocytopenia nor coagulopathy, swollen, sore joints (arthritis), abdominal pain, and renal involvement. IgAV can present at any age, even during adulthood, but the incidence is higher in children. The age at peak incidence is approximately four to six years old. Most cases in children present under the age of 10 years and are very rare in infancy. In the pediatric age group, the ratio of male-to-female presentation is 1.5:1. It also shows a decreasing incidence with increasing age [2]. Older children, mainly teenagers with IgAV, tend to have a disease similar to adult-onset IgAV [3]. Adult-onset disease rarely presents with abdominal involvement, but joint involvement is more predominant [4].

## Case presentation

We present a case of a 72-year-old man with a past medical history of osteoarthritis, type 2 diabetes mellitus, elbow fracture, hypertension, hyperlipidemia, total bilateral knee replacement, burns and, benign prostatic enlargement who presented to the emergency room because of foul-smelling wounds and bilateral leg swelling lasting three months, with the left leg being worse than the right. The patient was very weak, unable to walk, and needed the help of a wheelchair at presentation. He also reported shortness of breath with exertion, nausea, vomiting, vague abdominal pain with no tenderness nor guarding. He reported a 16-pound weight loss in a two-week duration. He had decreased appetite, dry heaves, and difficulty urinating despite taking a diuretic. Prior to this presentation, he was receiving treatment at an outpatient facility for the leg swelling with amoxicillin and furosemide. The edema has been intermittent, but it later became worse and necessitated his presentation to the hospital. He denied any history of fever, hematuria, symptoms of upper respiratory tract infections, or current smoking history. There has been no recent change in his medications. His significant examination findings were somnolence, bitemporal wasting, and multiple ecchymoses on his hips and thighs bilaterally. He had significant pitting edema and ulcerations on both legs.

His vital signs were within reference range, except his oxygen saturation at 94%. His complete blood count showed leukocytosis. A complete metabolic panel showed hyponatremia, hypokalemia, elevated creatinine, and deranged estimated glomerular filtration rate (GFR). The liver function test findings were within the reference range (Table 1). Urinalysis findings were unremarkable, and hepatitis B and C screens were non-reactive.

**Table 1:** Laboratory findings showing neutrophilia, leukocytosis, and electrolyte abnormalities *Outside of reference range. L, low; H, high; WBC, white blood cell; RBC, red blood cell; ESR, erythrocyte sedimentation rate; BUN, blood urea nitrogen; eGFR, estimated glomerular filtration rate; AST, aspartate transaminase; ALT, alanine aminotransferase.

Hematologic panel	Patient’s Values	Reference Range Values
WBC	13.2 x10^9^/L (*H)	4.5-11.0 x10^9^/L
RBC	4.81 x10^12^/L	4.3-5.9 x10^12^/L
Hemoglobin	13.1 g/dL (*L)	13.5-17.5g/dL
Platelets	431 x10^9^ /L	150-450 x10^9^ /L
Differential		
Neutrophil	89.1% (*H)	54%-62%
Absolute neutrophil count	11.8 neutrophils/mcl (*H)	1.5-8.0 neutrophils/mcl
Acute phase reactants		
ESR	38 mm/h (*H)	0-15 mm/hr
Basic Metabolic Panel		
Sodium	132 mEq/L (*L)	136-145 mEq/L
Potassium	3.3 mEq/L (*L)	3.5-5.0 mEq/L
Chloride	88 mEq/L (*L)	95-105mEq/L
Carbon dioxide	34.0 mEq/L (*H)	23-28 mEq/L
Anion Gap	10.0 mEq/L	>10 mEq/L
BUN	90 mg/dL (*H)	8-20 mg/dL
Creatinine	1.4 mg/dL (*H)	0.6-1.2 mg/dL
EGFR	50 mL/min/1.73m^2 ^(*L)	> 90 mL/min/1.73m^2^
Calcium	7.8 mg/dL (*L)	8.4-10.2 mg/dL
Glucose	183 mg/dL (*H)	40-70 mg/dL
Magnesium	2.5 mEq/L (*H)	1.5-2 mEq/L
Liver Function Test		
Alkaline Phosphate	43 U/L	30-100U/L
Total Protein	5.8 g/dL(*L)	6.0-7.8 g/dL
Albumin	2.6 g/dL(*L)	3.5-5. 5 g/dL
AST	22 U/L	8-40 U/L
ALT	14 U/L	8-40 U/L

A preliminary assessment of bilateral lower extremity cellulitis and possible lower extremity venous insufficiency was made. The patient was admitted and started on intravenous ampicillin/sulbactam 3 g every six hours. He received fluid replacement therapy to correct his electrolyte imbalance and dehydration. Wound care was given, and furosemide continued.

Echocardiography showed a healthy ejection fraction of 55%-60%. Doppler ultrasound of the lower extremities was negative for deep vein thrombosis. An autoimmune screen revealed elevated IgA levels. The anti-neutrophilic antibody screen was negative. Complement-3 (C3) was normal, Sjogren syndrome (SS)-A and SS-B were normal as well as nuclear ribonucleoprotein (RNP) antibody. Sclerodermal (Scl) 70, Jo-1, Smith antibody, ribosomal antibody, RNP, centromere antibody, double-stranded DNA antibody. Purified protein derivative test was negative.

**Figure 1:**
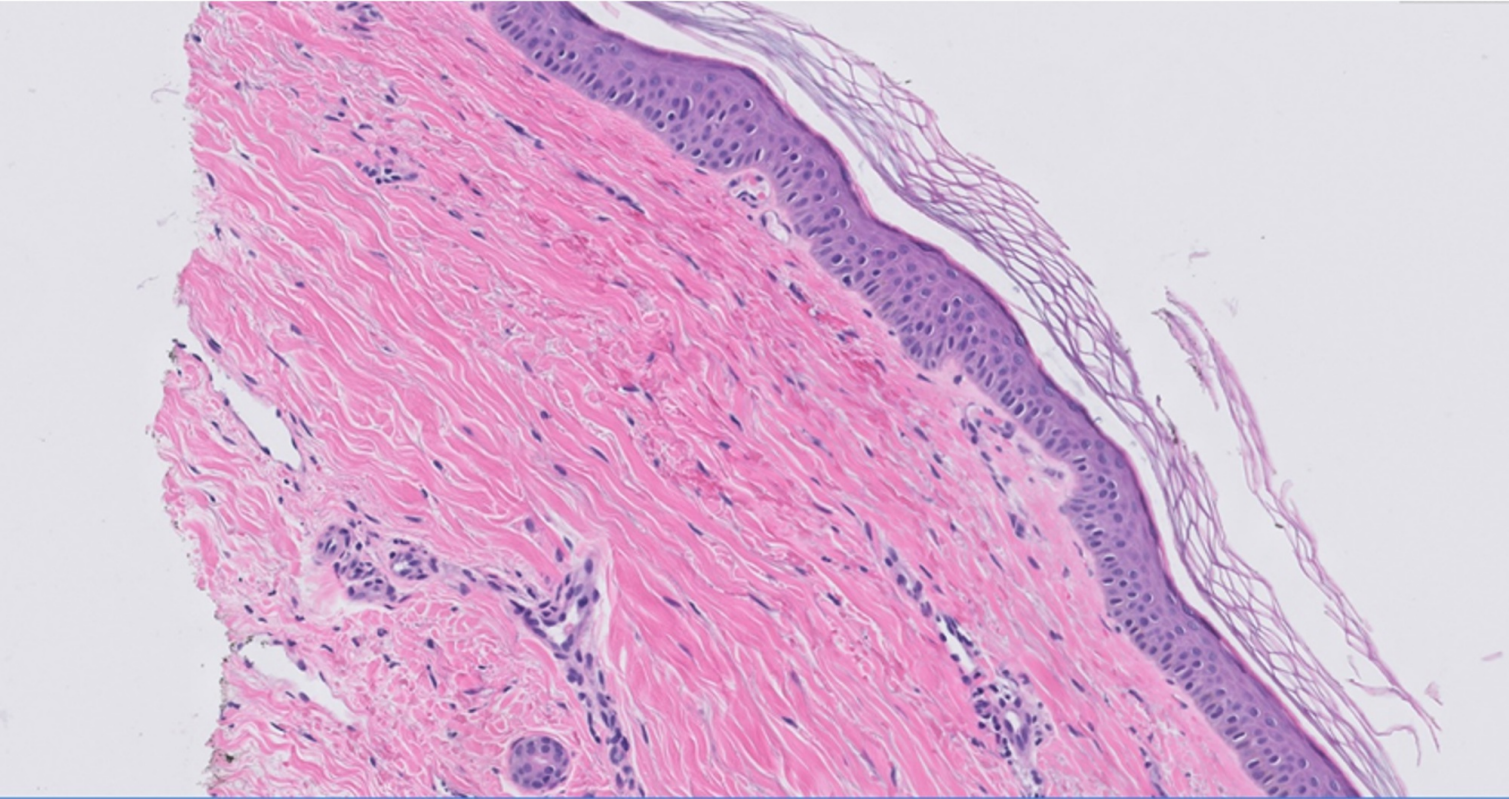
Medium power slide showing extravasated RBCs in the collagen; the vessels show some intimal thickening which reflects the IgA deposits RBC, red blood cells; IgA, immunoglobulin A.

A computed tomography scan of the abdomen showed inflammatory changes, wall thickening, and surrounding edema of proximal jejunum and distal duodenum on the small intestine. A gastrointestinal endoscopy with biopsy showed signs of vasculitis, necessitating a skin biopsy of the lower extremities, which was consistent with acute small vasculitis with IgA and C3 present (Figure 1). The patient was placed on oral prednisone 10 mg, three times daily. He was discharged home in a stable condition. He was seen for follow-up after discharge, and the lower extremity edema had significantly improved.

## Discussion

IgAV is a form of vasculitis that affects the small blood vessels of the entire body, mediated by the deposition of IgA complexes in the walls of the small blood vessels. It manifests with systemic symptoms, mainly involving the skin, gastrointestinal tract, kidneys, and joints [5,6]. The American College of Rheumatology diagnostic criteria for IgAV include clinically palpable purpura, which is a characteristic finding, age of disease onset at younger than 20 years, the presence of gastrointestinal symptoms, and histopathological evidence of inflammation, mainly of small blood vessels in the affected areas. Histopathological findings of leukocytoclastic vasculitis on Hematoxylin and Eosin staining and deposits of IgA immune complexes on immunohistochemical staining are also a diagnostic criterion [6]. IgAV is diagnosed when at least two of these four criteria are met. Our patient met two of the above criteria, which included gastrointestinal involvement and elevated IgA deposits in a skin biopsy. However, he had an unusual skin manifestation of cellulitis and signs of venous insufficiency with normal Doppler ultrasound imaging. Immunohistochemical staining of skin biopsy specimens showed IgA deposits in the walls of the blood vessels. This makes our case a unique one as it provides more insight into an unusual presentation and represents diagnostic challenges in the management of IgAV.

The disease distribution showed that children are more commonly affected than adults with a male-to-female ratio of 1.5:1 [2]. There are differences in the expression of the disease across the different age groups. Children were noted to have a preceding upper respiratory tract infection, fever, and abdominal pain, while adults had mostly symptoms of joint pain and increased sedimentation rates, and more frequent and severe renal involvement [4,7].

There are two broad categories of IgAV, namely IgAV without kidney involvement and IgA nephritis and nephropathy. IgA nephritis ranges from asymptomatic hematuria to one of full-blown nephrotic or nephritic syndrome [8]. The pathogenesis of IgAV with both nephropathy and nephritis is linked to abnormal O-glycosylation of serum IgA1 and its deposition into the renal mesangium. Of note, IgAV without kidney involvement lacks this abnormal O-glycosylation [8]. The prognosis of IgAV in both adults and children is usually benign and self-limited, with complete recovery in 93.9% of the children and 89.2% of the adults [7]. However, poor prognostic factors are mainly older age of onset (older than 50 years), severe renal impairment, macroscopic hematuria, and persistent proteinuria.

Furthermore, the severity of the initial renal presentation is a critical determinant in regards to long term prognosis whereby the risk of progression to end-stage renal disease is less than 5% when initial signs at presentation are only hematuria and/or minimal proteinuria compared to 50% when both nephritic and nephrotic syndromes are present. More so, gastrointestinal and renal involvement are the significant causes of morbidity and mortality. Patients with no proteinuria in IgAV present with low serum albumin due to loss via the intestine. Timely intervention can prevent gastrointestinal complications such as perforation, hemorrhage, and necrotizing enterocolitis [5,9]. Pulmonary hemorrhage and cardiac complications are rare complications that mainly occur in adults and are associated with high morbidity and mortality [10,11]. Our patient's laboratory findings showed deranged GFR with electrolyte imbalances likely due to dehydration. These abnormalities were corrected with fluid replacement therapy.

The management of IgAV starts with a detailed history taking, physical examination, and relevant investigation, including biopsy of the primary involved organ [12]. Different immunosuppressants such as corticosteroids, cyclophosphamide, azathioprine, and rituximab are effective in the treatment of IgAV [5,13]. Early prednisone therapy has been shown to be beneficial in patients with extra renal symptoms. Initiation of prednisone reduces both severity and duration of abdominal pain during the first two weeks of treatment [14]. Also, studies have shown that prednisone mostly helps in alleviating the symptoms and does not necessarily change the clinical course and the chances of recurrence after therapy [14].

## Conclusions

IgAV is a rare disease in adults. Also, the presence of cutaneous and abdominal findings are rare manifestations in adults with IgAV. Clinicians should suspect a case of IgAV in an adult presenting with lower extremity lesions and edema with negative Doppler ultrasound findings. Even though the prognosis of IgAV is usually favorable, it is important for clinicians to diagnose and initiate appropriate treatment promptly to avoid severe complications.
